# Mesoscopic Modeling of the Encapsulation of Capsaicin by Lecithin/Chitosan Liposomal Nanoparticles

**DOI:** 10.3390/nano8060425

**Published:** 2018-06-12

**Authors:** Ketzasmin A. Terrón-Mejía, Evelin Martínez-Benavidez, Inocencio Higuera-Ciapara, Claudia Virués, Javier Hernández, Zaira Domínguez, Waldo Argüelles-Monal, Francisco M. Goycoolea, Roberto López-Rendón, Armando Gama Goicochea

**Affiliations:** 1 Centro de Investigación y Asistencia en Tecnología y Diseño del Estado de Jalisco, A.C., Av. Normalistas 800, Colinas de la Normal, Guadalajara 44270, Mexico; ket.a.t.m@gmail.com (K.A.T.-M.); emartinez@ciatej.mx (E.M.-B.); inohiguera@ciatej.mx (I.H.-C.); 2 Instituto Tecnológico Superior de Zongolica, Km. 4 Carretera a la Compañía, Zongolica, Veracruz 95005, Mexico; 3 Centro de Investigación y Asistencia en Tecnología y Diseño del Estado de Jalisco, A.C., Clúster Científico y Tecnológico Biomimic^®^, Carretera antigua a Coatepec No. 351, Colonia El Haya, Xalapa, Veracruz 91070, Mexico; cvirues@ciatej.mx; 4 Unidad de Servicios de Apoyo en Resolución Analítica, Universidad Veracruzana, Apartado Postal 575, Xalapa, Veracruz 91190, Mexico; javmartinez@uv.mx (J.H.); zdominguez@uv.mx (Z.D.); 5 Centro de Investigación en Alimentación y Desarrollo A. C., Grupo de Investigación en Biopolímeros, Carr. a La Victoria km. 0.6, Hermosillo 83304, Mexico; waldo@ciad.mx; 6 School of Food Science and Nutrition. University of Leeds. Woodhouse Ln, Leeds LS2 9JT, UK; F.M.Goycoolea@leeds.ac.uk; 7 Laboratorio de Bioingeniería Molecular a Multiescala, Facultad de Ciencias, Universidad Autónoma del Estado de México, Av. Instituto Literario 100, Toluca 50000, Mexico; 8 División de Ingeniería Química y Bioquímica, Tecnológico de Estudios Superiores de Ecatepec, Av. Tecnológico s/n, Ecatepec 55210, Mexico

**Keywords:** capsaicin, chitosan, lecithin, dissipative particle dynamics

## Abstract

The transport of hydrophobic drugs in the human body exhibits complications due to the low solubility of these compounds. With the purpose of enhancing the bioavailability and biodistribution of such drugs, recent studies have reported the use of amphiphilic molecules, such as phospholipids, for the synthesis of nanoparticles or nanocapsules. Given that phospholipids can self-assemble in liposomes or micellar structures, they are ideal candidates to function as vehicles of hydrophobic molecules. In this work, we report mesoscopic simulations of nanoliposomes, constituted by lecithin and coated with a shell of chitosan. The stability of such structures and the efficiency of the encapsulation of capsaicin, as well as the internal and superficial distribution of capsaicin and chitosan inside the nanoliposome, were analyzed. The characterization of the system was carried out through density maps and the potentials of mean force for the lecithin-capsaicin, lecithin-chitosan, and capsaicin-chitosan interactions. The results of these simulations show that chitosan is deposited on the surface of the nanoliposome, as has been reported in some experimental works. It was also observed that a nanoliposome of approximately 18 nm in diameter is stable during the simulation. The deposition behavior was found to be influenced by a pattern of N-acetylation of chitosan.

## 1. Introduction

The advent of nanobiotechnology has seen increasing research and development in the use of bioconjugates as new therapeutic alternatives [1]. One of the main goal of these technologies is to focus on utilizing inherent structural, specific recognition or the catalytic properties of biomolecules to assemble either composite nanoscale materials or devices with unique or novel properties [2]. For instance, several carriers, comprised of biopolymers, macromolecules, and liposomes, have been used to deliver drugs in vivo [3]. Liposomes are microscopic vesicles formed essentially by phospholipids, which are amphiphilic molecules containing polar heads and hydrophobic hydrocarbon tails that have the ability to self-associate spontaneously and form bilayer vesicles, dispersed in water [4]. Nanoarchitectonics has emerged as a new way to create functional materials at the nano level [5,6,7,8,9]. Taking elements from nanotechnology and supramolecular chemistry, nanoarchitectonics guides the arrangement of nanoscopic unit components into specific configurations, in order to obtain higher order functional materials [10]. Harmonization of various phenomena and processes (including atomic and molecular manipulation, self-assembly, and organization) is required to achieve this, since nano objects are under quantum effects and are affected by interactions with other molecules and by thermal/statistical fluctuations. In recent years, several functional materials have been synthesized following the nanoarchitectonics concept that explore multiple applications, including those that belong to the fields of biology and biomedicine [11]. In this context, the design of materials with drug delivery functions is undoubtedly a promising application of nanoarchitectonics, as has been reported in the literature [5,6,7,8,9]. The understanding of the way in which the components of nanocarriers interact with each other is an important part of the successful design of this type of material.

The use of colloidal carriers made of hydrophilic polysaccharides, i.e., chitosan (CS), has arisen as a promising alternative for improving the transport of therapeutic peptides, proteins, oligonucleotides, and plasmids across biological surfaces [12]. CS is the common name of a linear, random copolymer of β-(1–4)-linked d-glucosamine and *N*-acetyl-d-glucosamine, whose molecular structure is comprised of a linear backbone linked through glycosidic bonds. CS is a hydrophilic, biocompatible, and biodegradable polymer of low toxicity. Recent reviews have highlighted the potential use of CS-based drug deliverers [13,14]. Also, the characteristics of CS-coated liposomes and their interactions with leuprolide have been investigated by Gou et al. [15].

On the other hand, lecithin, which has two long hydrocarbon chains, is a major component of lipid bilayers of cell membranes and is a natural, biological amphiphile. Lecithin is a natural lipid mixture of phospholipids, and is frequently used for the preparation of various nanosystem delivery vehicles, such as microemulsions, liposomes, micelles, and nanoparticles; it is considered to be a safe and biocompatible excipient [16,17,18]. The potential of the various applications that have been found in lecithin/CS nanoparticles has already been reported—for instance, their potential to be a mucoadhesive colloidal nanosystem for transmucosal delivery of melatonin was investigated [19], or as a topical delivery system for quercetin [20]. The encapsulation of quercetin into lecithin/CS nanoparticles [21] and chitosan-coated nanocapsules [22], as well as the influence of loaded tamoxifen on the structure of lecithin/CS nanoparticles, has also been investigated [23]. Other nanostructured materials that harness the interactions of lecithin phospholipid/CS have comprised electrospun nanofibers [24] and nanoporous hydrogels [25].

Capsaicin (8-methyl-*N*-vanillyl-6-nonenamide), a lipophilic drug, is the pungent vanilloid compound in spicy chili peppers. It is also approved as a drug for the treatment of chronic pains (e.g., arthritis, migraine, and diabetic neuropathy) [26,27], and its potential use in the treatment of urological disorders, as well as the control of satiety and obesity [28], has also been documented. Capsaicin is known to be an agonist of the transient receptor potential vanilloid 1 (TRPV1), a nonselective cation channel involved in the detection of body temperature and heat nociception [29]. Capsaicin has a nociceptive-blocking action that is the basis of its pharmacological use as an analgesic in persistent pathological pain states [30].

In light of the well-established pharmacological activities of capsaicin, identifying new potential nanocarriers for intracellular, transdermal, and intranasal delivery of capsaicin, so as to regulate the activity of its receptors in various tissues, is appealing as a potential emerging therapeutic strategy. However, the handling and administration of capsaicin is not always feasible, due to its pungency, cytotoxicity at high concentrations [31], and sparing solubility in water [32]. Several studies have been conducted to incorporate capsaicin into nanoformulations, in an attempt to make it more compatible with aqueous physiological environments [33,34]. For instance, capsaicin-loaded nanoemulsions stabilized with a natural biopolymer like alginate or CS has been used as a functional ingredient delivery system [35]. In a few studies, we have encapsulated capsaicin using oil-core CS-based nanocapsules, and examined the effect on modulating capsaicin’s pungency [34,36]. The drug encapsulation efficiency determined in oil-core nanoemulsions (uncoated) was ~50% [36], while in chitosan-coated nanocapsules, this value ranged from ~66% to 92% [36], and seemed to increase with the molecular weight of chitosan [36]. The drug-loading capsaicin of these formulations was 2% and the presence of capsaicin did not have a significant influence on the size, ζ-potential, or morphology.

Notwithstanding the aforementioned, the use of nanostructured materials or functionalized nanocarriers with biopolymers has received a great deal of attention. Recent studies show that biopolymers, such as CS or cellulose and modifications of them, are ideal candidates for developing improved nanocapsules [28,29], with increased efficiency in the delivery of different drugs, mainly of the hydrophobic type [30]. These drugs have very low solubility, so their biodistribution is very biopolymers, such as CS or cellulose and modifications of these, are ideal candidates for developing improved nanocapsules [37,38,39] with increased efficiency in the delivery of different drugs, mainly of limited if there is no suitable vehicle for administration. Thus, nanocapsules represent a very good option to solve this problem. In addition, colloidal nanocapsules with an oily core and a CS shell have been investigated as potential nanocarriers for transmucosal drug delivery [40]. These systems assemble by spontaneous emulsification [41,42], and are versatile because they can carry both lipophilic and hydrophilic macromolecules [43,44].

Experimental work [45,46] has shown compatibility between lecithin/CS liposomal nanoparticles as nanocarriers. Coated liposomes may subsequently be of significant interest to food and pharmaceutical industries for the improved delivery of lipophilic and hydrophilic functional components, such as flavors, antioxidants, antimicrobials, and bioactives [46]. However, at the molecular level it has been difficult to elucidate the nature of the interactions between these components in order to optimize the formation of nanocapsules. On the other hand, one can dispose of the methods of computational modeling that are available to study such interactions [47,48]. These tools have been shown to be quite effective in the prediction of physicochemical and structural properties of complex systems [49,50]. Thus, the goal of the current work was to use mesoscopic simulations, through the approach of dissipative particle dynamics (DPD), to analyze and characterize nanocapsules constituted by nanoliposomes of lecithin coated with a shell of CS and their function as nanocarriers for the administration of capsaicin, which has proven applications in some therapies.

The remainder of this paper is organized as follows. The models and methods are detailed in Section 2. The results and discussion are presented in Section 3. Finally, the conclusions are drawn in Section 4. The general equations of the DPD approach, the simulation methodology, and full details of our models presented in this work can be found in the supplementary information (SI) that accompanies this paper.

## 2. Models and Methods

**Figure 1:**
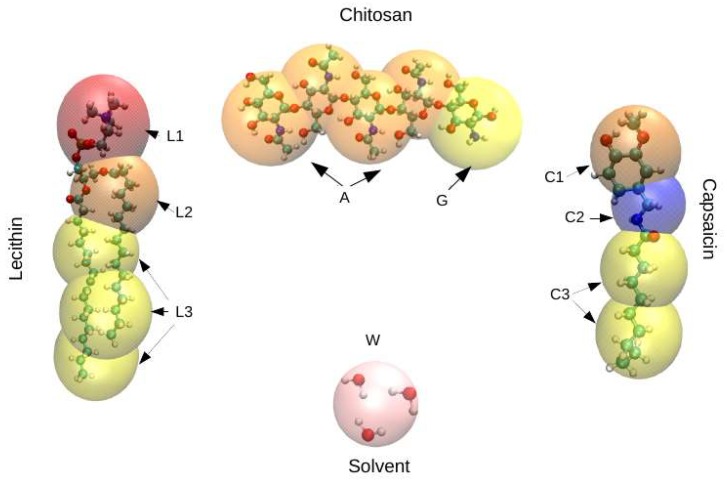
Schematic representation of the coarse-grained models adopted in this work. Mesoscopic models for lecithin (C_42_H_82_NO_8_P, **left)**, chitosan (CS) (**top center**), water as solvent (**bottom center**), and capsaicin (C_18_H_27_NO_3_, **right**). The exact division of every functional group is presented in the SI. As an overview, the molecular structure of lecithin is composed of three different beads, which we have labelled as *L*1, *L*2, and *L*3, that correspond to the head, neck and tail groups, respectively. The same nomenclature is used for capsaicin, where the beads *C*1, *C*2, and *C*3 correspond to the head, neck, and tail groups, respectively. The CS model consists of two types of beads: the first bead represents the glucosamine units (C_6_H_13_NO_5_), which are labelled *G*, while the second bead represents the *N*-acetyl-glucosamine units (C_8_H_15_NO_6_), which are labelled *A*. Finally, the solvent (water) is represented by bead *W*. These figures were prepared with the Visual Molecular Dynamics (VMD) package [53].

DPD is a mesoscopic technique previously shown to be successful in the prediction of equilibrium and non-equilibrium properties of soft matter systems [50,51], which makes it suitable to study the coating of nanoliposomes by CS. The numerical simulations presented in this work were carried out via the canonical ensemble (constant density and temperature), with the global density set equal to three, as is usually done. Molecules of lecithin, capsaicin, and CS were derived from the atomic structure in a coarse-grained molecular model, as show in Figure 1. For practical purposes, the particles in the coarse-grained models are interpreted as groups of atoms instead of individual atoms. The details of this parametrization are in the SI that accompanies this document. All simulations reported here were performed with our software, SImulation at MESoscopic scale (SIMES) [52], which is designed to run simulations completely on graphic processing units (GPUs) under the DPD framework.

## 3. Results and Discussion

**Figure 2:**
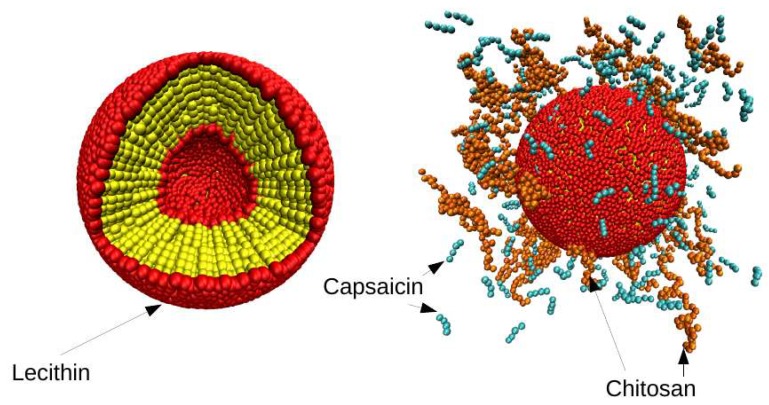
Initial configuration of nanoliposome. A snapshot of the initial configuration of lecithin molecules in the structure of a liposome bilayer (**left**). The yellow spheres represent the hydrophobic part of the lecithin, while the red spheres represent the hydrophilic part. The capsaicin and CS molecules were placed in a random configuration around the lecithin (**right**). The orange chains represent the CS polymers, while the turquoise chains represent the capsaicin molecules. These Figures were prepared with the VMD package [53].

The simulations were performed starting from an initial configuration of the liposome as shown in Figure 2 (left). This liposome was found in aqueous solution together with capsaicin molecules and polymeric chains of CS, which were dispersed in a random configuration, as shown in Figure 2 (right). The simulations carried out in this work were divided into two sets: the first one consisted of keeping the concentration of capsaicin fixed and exploring the behavior of the nanocapsule as a function of CS concentration in solution. The second set involved CS, and we explored the influence of the degree of deacetylation (DA) by changing the polymer sequence, as is described below. The degree of deacetylation represents the proportion of glucosamine units (deacetylated monomers) in a CS polymer molecule. The DA of CS is an important characterization parameter, since it influences several physicochemical properties [54,55]. For this second case, the distribution of *N*-acetyl-d-glucosamine (GlucNA) and d-glucosamine groups (GlcN), over the CS chain affects the properties of the polymer. It also allows the appearance of cooperative effects, due to the association of hydrophobic units with the concomitant effect of the properties of nanocapsules. To achieve this purpose, we explored two different sequences (S1 and S2) in the CS polymer, finding that the first (S1) was more random that the second (S2). Both sequences maintained a degree of *N*-acetylation of 30%. The main criterion to select such a value of *N*-acetylation was based on our previous experimental studies, in which we found that chitosan chains with intermediate values of *N*- acetylation are more effective at delivering microRNA intracellularly [56], and interact more efficiently with mucin due to a greater conformational adaptation [57]. Also, this degree of *N*-acetylation corresponds well with that of experimental chitosan molecules, with a non-random pattern of acetylation being developed in ongoing studies in our laboratories.

The CS monomer sequences considered in the simulations were prepared as follows:

(a) ${\left[-\mathrm{GlucNA}-{\left[\mathrm{GlcN}\right]}_{3}-\mathrm{GlucNA}-{\left[\mathrm{GlcN}\right]}_{3}-\mathrm{GlucNA}-\mathrm{GlcN}-\right]}_{5}$
(b) ${\left[-{\left[\mathrm{GlucNA}\right]}_{4}-{\left[\mathrm{GlcN}\right]}_{9}\right]}_{3}-{\left[\mathrm{GlucNA}\right]}_{3}-{\left[\mathrm{GlcN}\right]}_{8}$

According to this nomenclature, the first sequence consists of five blocks. Each of these blocks was composed of a unit of GlucNA, followed by three units of GlcN. These two sections are repeated, and the sequence ends with a GlucNA unit linked to GlcN. The second sequence is composed of three sections. The first section consists of three blocks, which are formed by four units of GlucNA bonded to nine units GlcN. The second section contains three units of GlucNA, while the third section contains eight units of GlcN.

### 3.1. Influence of Chitosan Concentration on Lecithin

**Figure 3:**
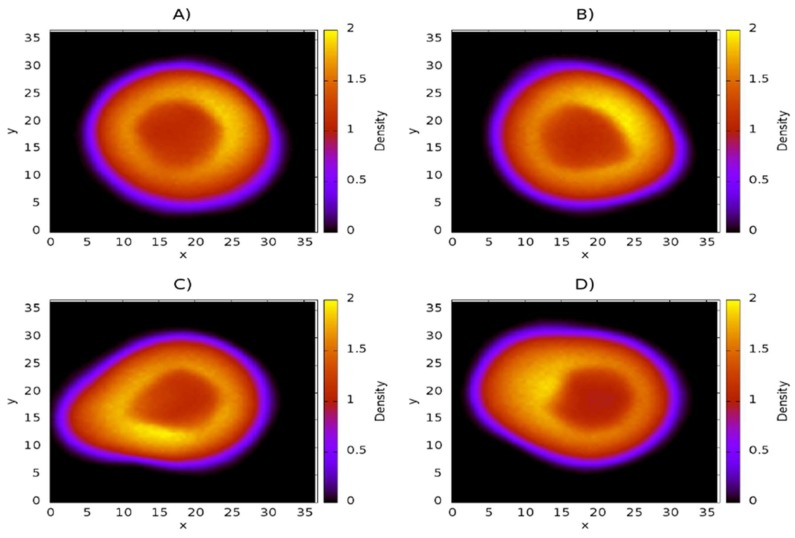
Density maps of lecithin on the *xy* plane at different concentration of CS. (**A**) 50 chains of CS (6 mM); (**B**) 100 chains of CS (12 mM); (**C**) 150 chains of CS (18 mM); (**D**) 200 chains of CS (24 mM). The scale of density bars starts at 0.0 (black regions) and reaches a maximum of 2.0 (yellow regions). All quantities are expressed in reduced dissipative particle dynamics (DPD) units.

Density maps corresponding to lecithin, CS, and capsaicin were obtained from the simulations. These density maps, mainly of lecithin, are useful for determining if the structure of the liposome was affected by the presence of CS or capsaicin. First, the concentration of capsaicin in the system (250 molecules of capsaicin) was fixed, and then the concentration of CS was changed, starting with 50 chains up to a total of 200 chains, in increments of 50 chains. These amounts of CS correspond to concentrations of 6, 12, 18, and 24 mM, respectively. The density maps of lecithin as a function of CS concentration are shown in Figure 3. They show the structure of the liposome, as well as the region formed by the lipid membrane, which is presented as the region with the highest concentration (in yellow) in the four cases. From these maps, it is possible to observe that the core zone shows a more reddish tone, which indicates that the lipid density is lower, as expected. Density maps also show that the nanoliposome is stable during 24 μs of simulation, since the lecithin molecules do not spread all over the simulation box, nor do they collapse in the aqueous core to form a micelle.

In regard to the increase of CS in the system, Figure 3A shows that the structure of the nanoliposome is not affected by CS; the nanoliposome remains quasi spherically symmetrical. Figure 3B shows that the nanoliposome undergoes minimal alteration in its structure, losing some spherical symmetry. Figure 3C shows a pronounced protuberance in the nanoliposome, located in the interval (5, 10) along the *x* coordinate. In this case, the lipid membrane could break, thus influencing the structural stability of the nanoliposome. In Figure 3D, a protuberance similar to that shown in Figure 3C occurs. Under these conditions, the liposome membrane is thicker.

### 3.2. Distribution of Chitosan on Liposome

**Figure 4:**
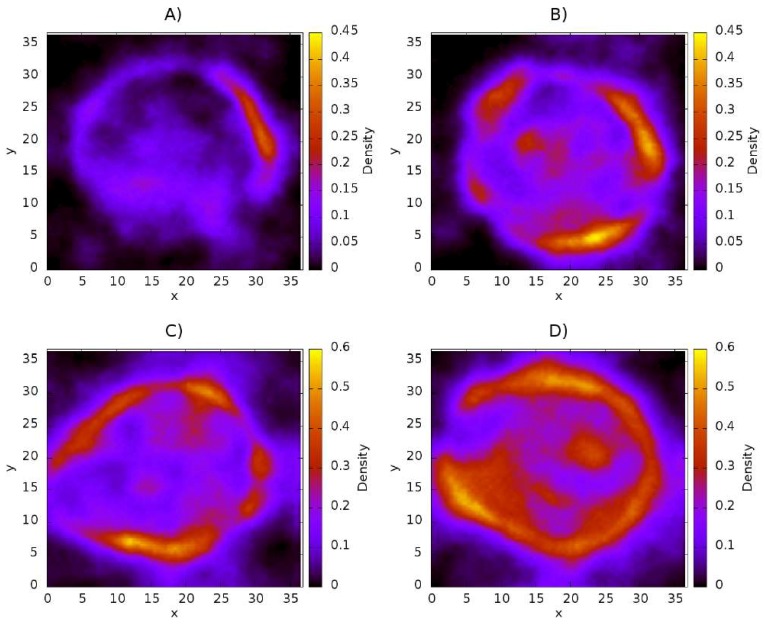
Distribution of CS on liposome on the *xy* plane. (**A**) 50 chains of CS; (**B**) 100 chains of CS; (**C**) 150 chains of CS; (**D**) 200 chains of CS. All quantities are reported in reduced DPD units.

The density maps corresponding to CS are shown in Figure 4. These maps show only the density of the molecule in question (in this case CS) on the *xy*–plane, with the scale shown on the right side of every panel. It is a three-dimensional representation of the spatial distribution of the CS inside the liposome. The panels A–D show the CS distribution as its concentration is increased from 50 CS chains (Figure 4A) to 200 CS chains (Figure 4D). These maps show that CS is adsorbed over the surface of the nanoliposome, as has been reported in experimental work [58,59,60]. It is also possible to see how the surface becomes more homogeneous with increasing CS concentration. Figure 4A shows that the CS concentration is not sufficient to cover the surface of the nanoliposome, since the area comprising the radius of the nanoliposome exhibits regions of very low density, which indicates a deficiency of polymeric chains in the zone. Figure 4B shows that increasing the concentration by 50 CS chains, equivalent to a concentration of 12 mM, results in the nanoliposome becoming uniformly coated by the polymer. The appearance of yellow regions are indicative of an association of CS polymers. Figure 4C shows the distribution of CS at a concentration of 18 mM on the liposome. The regions of greater density are more pronounced than in the previous case. A greater adsorption of CS is observed too. In this case, the polymer acts as a protective layer. The last case represents the adsorption of polymers of CS at a concentration of 24 mM (Figure 4D). Under this condition, the surface of the liposome becomes almost completely coated. It is also possible to observe how an increasing concentration of CS raises the quantity of a supernatant polymer in an aqueous medium, thus indicating that a competitive association between CS-liposome and CS-CS is present. The blue regions refer to a low density of CS, while the red regions refer to regions of high CS density.

### 3.3. Influence of Chitosan Concentration on Capsaicin

**Figure 5:**
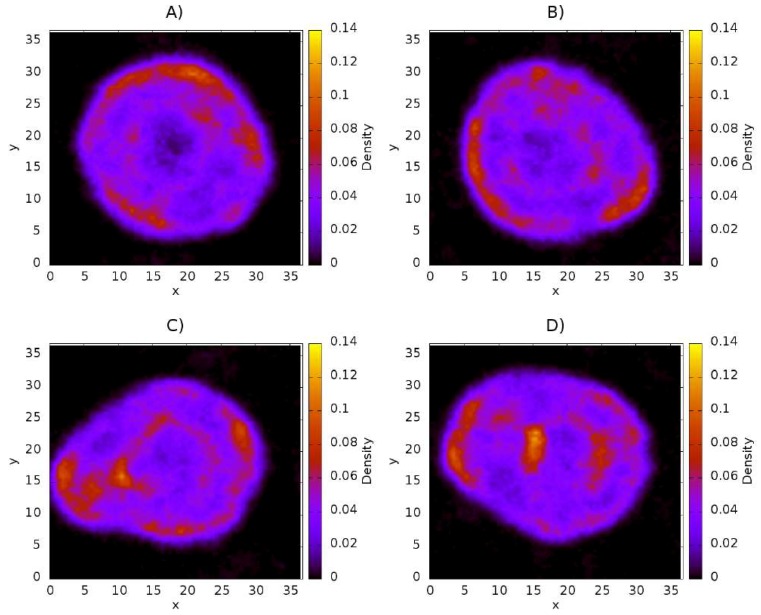
Influence of CS concentration on capsaicin on the *xy* plane at different concentrations of the CS polymer. (**A**) 50 chains of CS; (**B**) 100 chains of CS; (**C**) 150 chains of CS; (**D**) 200 chains of CS. All quantities are reported in reduced DPD units.

Density maps corresponding to the capsaicin molecules are shown in Figure 5. These maps clearly show that capsaicin is absorbed and encapsulated in the nanoliposome. This phenomenon is not affected by the presence of the CS polymer, which indicates that interactions between capsaicin and CS are very weak in comparison to the interactions between lecithin and capsaicin. In Figure 5A–D it is possible observe how the capsaicin molecules are deposited close to the interface between the nanoliposome and the aqueous medium, and even in the interface with the aqueous core, which suggests that capsaicin will be transported by the oil phase in the nanoliposome, thus leaving free the aqueous core with the capacity to transport other hydrophilic molecules with therapeutic potential. If the capsaicin reached the core, a yellow or high-density region would be observed in the center, which does not occur.

### 3.4. Potentials of Mean Force

**Figure 6:**
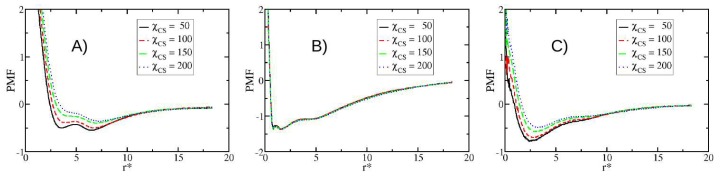
Potentials of mean force (PMF) for different concentration of CS, as function of separation distance between mass centers of each molecule. (**A**) Lecithin-CS; (**B**) lecithin-capsaicin; and (**C**) capsaicin-CS. All quantities are expressed in reduced DPD units.

Other properties obtained from simulations are the potentials of mean force (PMF), which are many-body interactions arising from their complex interplay beyond mean-field approximations [61]. The details about the calculation of the PMF can be found in the SI. Figure 6 shows the PMF between lecithin-CS, lecithin-capsaicin, and capsaicin-CS. At higher concentrations of CS, the interaction becomes weaker, indicating that adsorption over the surface of the nanoliposome decreases when more CS molecules are in solution. Figure 6B corresponds to the PMF of lecithin-capsaicin. This shows that the interaction between capsaicin and the nanoliposome is not significantly affected by the presence of CS chains, since their PMF are practically the same, making it clear that the interactions between CS and capsaicin are not the leading mechanism of nanocapsule conformation. Figure 6A,B show two minimal values that are attributed to the lipid bilayer. Figure 6C shows the PMF between capsaicin and CS. It is evident that the attractive interactions become weaker as the quantity of CS increases. This is due to the presence of competitive adsorption, which promotes the self-association between CS molecules, so that the interactions between polymer chains with a nanoliposome surface, as well as those with capsaicin molecules, become weaker, thus causing deposition mainly in the surface of the nanoliposome.

### 3.5. Modifying the Sequence of Chitosan

**Figure 7:**
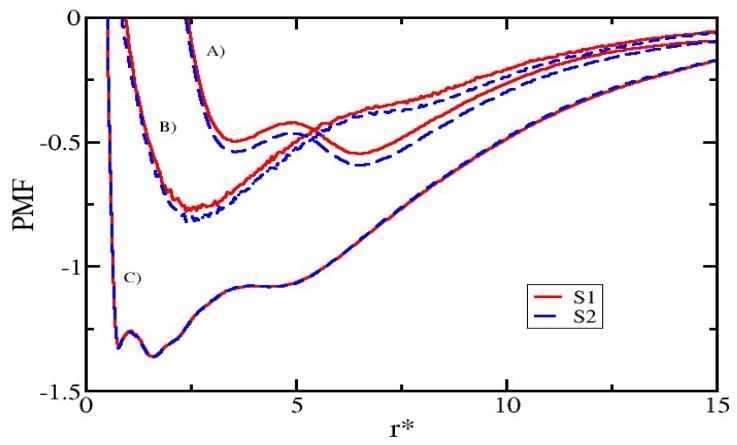
PMF for the two sequences of CS used in this work, S1 (red line) and S2 (blue dotted line), to concentrations of CS reaching 6 mM. PMF for (**A**) lecithin-CS; (**B**) CS-capsaicin; and (**C**) lecithin-capsaicin. All quantities are expressed in reduced DPD units.

In this section, we present the results obtained from the changes in the CS sequence pattern composition. The analysis presented below corresponds to a second group of simulations described at the beginning of this section. The results show that the pattern (sequence) in which the GlucNA and GlcN monomers are arranged has a clear effect on the PMF (Figure 7). We note that the PMF corresponding to CS-lecithin and CS-capsaicin indicate a favourable condition for attractiveness in the case of sequence S2 with respect to sequence S1, indicating a slight presence of cooperative effects between GlucNA units, which promote the association of CS with the nanoliposome and hence with capsaicin. On the other hand, the interaction between lecithin and capsaicin is not affected or modified by these cooperative effects, so the interaction between capsaicin and CS remains weaker than the interaction of capsaicin with lecithin.

### 3.6. Mean Size of Nanoliposome and Encapsulation Efficiency (EE)

Additional properties obtained from the simulations are the size of the nanoliposome and the encapsulation efficiency of capsaicin. These properties are shown in Table 1. We obtained the encapsulation efficiency (EE) and mean size from the density profiles of capsaicin and lecithin, respectively, which are shown in the SI. EE is obtained from the equation EE = ((CapsT − CapsF)/CapsT) × 100, where CapsT is the total concentration of capsaicin in the system and CapsF is the free capsaicin in solution.

The mean size of the simulated nanoliposome is smaller but comparable to those obtained experimentally for CS-coated oil-core nanocapsules (~80–250 nm) [18,43,58,59]. This is an encouraging aspect of mesoscale simulation techniques, such as DPD. Additionally, the percentage of capsaicin encapsulation was very close (96%) to that reported experimentally (92%) [58].

**Figure 8:**
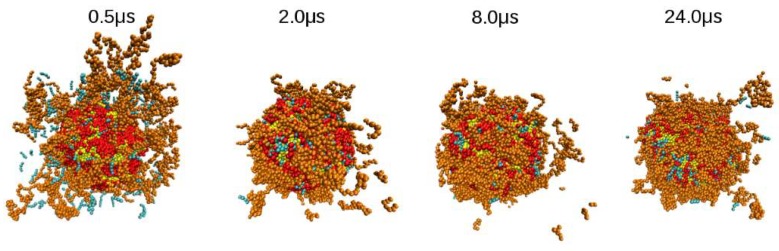
Snapshots of adsorption of CS on the nanoliposome at various times during simulation. In these pictures, the conformation of the nanocapsule along different times can be observed. The color code in this figure is the same as the one in Figure 2. The solvent molecules are not shown for clarity purposes. These figures were prepared with the VMD package [53].

Figure 8 shows snapshots obtained from the simulation trajectory, where the system was monitored over time. It can be observed that capsaicin is being encapsulated inside of nanoliposomes, while CS is deposited on the surface. An animation of this simulation is added in the SI section, where capsaicin encapsulation can be clearly observed.

## 4. Conclusions

We performed DPD simulations to analyze the stability of nanocapsules formed by nanoliposomes with a polyelectrolyte shell (CS). Results obtained from density maps showed that the nanocapsule is stable and is smaller than but comparable to nanocapsules experimentally obtained. The information provided by the potentials of mean force showed that the interaction between capsaicin and CS is very weak compared to that with lecithin. An association between capsaicin and CS in presence of lecithin is not likely to occur. Under experimental conditions, the solvent may harbor other molecular compounds that can reduce the absorption of capsaicin by the nanoliposome.

## Figures and Tables

**Table 1:** Mean size of the nanoliposome and encapsulation efficiency (EE) as a function of quantity of CS.

CS	Size (nm)	± (nm)	EE (%)	± (%)
50	17.89	0.61	96.80	0.59
100	17.95	0.46	96.27	0.69
150	18.04	0.95	96.28	0.51
200	17.90	1.00	96.71	0.61

## Supplementary Materials

The following are available online at http://www.mdpi.com/2079-4991/8/6/425/s1, the details of the methodology are included and description of models used in this work.
