# Supplementary material for: Mesoscopic Modeling of the Encapsulation of Capsaicin by Lecithin/Chitosan Liposomal Nanoparticles
Source: Nanomaterials (Basel). 2018 Jun 12;8(6):425. doi: 10.3390/nano8060425 (PMC6027167; doi:10.3390/nano8060425)
Supplement: Supplementary file 1 [file nanomaterials-08-00425-s001.pdf]

nanomaterials

MDPI

Supplementary Information

Mesoscopic Modeling of Encapsulation of Capsaicin by Lecithin/Chitosan Liposomal Nanoparticles

Ketzasmin A. Terrón-Mejía [1,2], Evelin Martínez-Benavidez [1], Inocencio Higuera-Ciapara [1], Claudia Virués [3], Javier Hernández [4], Zaira Domínguez [4], W. Argüelles-Monal [5], Francisco M. Goycoolea [6], Roberto López-Rendón [7]*, and Armando Gama Goicochea [8]*

[1] Centro de Investigación y Asistencia en Tecnología y Diseño del Estado de Jalisco, A.C., Av. Normalistas 800, Colinas de la Normal, Guadalajara, Jalisco, 44270, México; (K.A.T.-M. : ket.a.t.m@gmail.com), (E.M.-B. : emartinez@ciatej.mx), (I.H.-C. : inohiguera@ciatej.mx)

[2] Instituto Tecnológico Superior de Zongolica, Km. 4 Carretera a la Compañía, Zongolica, Veracruz, 95005, México; (K.A.T.-M. : ket.a.t.m@gmail.com)

[3] Centro de Investigación y Asistencia en Tecnología y Diseño del Estado de Jalisco, A.C., Clúster Científico y Tecnológico Biomimic®, Carretera antigua a Coatepec No. 351, Colonia El Haya, Xalapa, Veracruz 91070, México; (cvirues@ciatej.mx)

[4] Unidad de Servicios de Apoyo en Resolución Analítica, Universidad Veracruzana, Apartado Postal 575, Xalapa, Veracruz, 91190, México; (J.H. : javmartinez@uv.mx), (Z.D. : zdominguez@uv.mx)

[5] Centro de Investigación en Alimentación y Desarrollo A. C., Grupo de Investigación en Biopolímeros, Carr. a La Victoria km. 0.6, Hermosillo, Sonora, 83304, México; waldo@ciad.mx

[6] School of Food Science and Nutrition. University of Leeds. Woodhouse Ln, Leeds LS2 9JT, United Kingdom; F.M.Goycoolea@leeds.ac.uk

[7] Laboratorio de Bioingeniería Molecular a Multiescala, Facultad de Ciencias, Universidad Autónoma del Estado de México, Av. Instituto Literario 100, Toluca, Estado de México, 50000, México; roberto.lopez.rendon@gmail.com

[8] División de Ingeniería Química y Bioquímica, Tecnológico de Estudios Superiores de Ecatepec, Av. Tecnológico s/n, Ecatepec, Estado de México, 55210, México; agama@alumni.stanford.edu

* Correspondence: roberto.lopez.rendon@gmail.com; agama@alumni.stanford.edu; Tel.: +52-555-000-2300, and +52-722-296-5554

Received: date; Accepted: date; Published: date

Keywords: capsaicin; chitosan; lecithin; dissipative particle dynamics

I. METHODOLOGY, MODELS AND COMPUTATIONAL DETAILS

A. Dissipative particle dynamics simulation (DPD)

Some years ago Hoogerbrugge and Koelman [1] introduced a new simulation technique called dissipative particle dynamics (DPD). It is based on the simulation of soft spheres ("beads"), whose motion is governed by simple force laws; in addition, it allows for the mesoscopic–scale modeling of the self–assembly of surfactant and polymer systems. DPD is based on a coarse–grained representation, where the internal degrees of freedom of the molecules are integrated out in favour of a less atomistically detailed and more mesoscopic description of the system. Beads interact through soft, short range potentials that lead to improved computational efficiency. Despite the simplicity of the models, DPD can provide quantitatively and qualitatively correct descriptions of structural and thermodynamic properties of complex systems [2, 3].

DPD is an approach based on the classical equations of motion, DPD has enjoyed enormous popularity in the modeling of systems at mesoscopic scale. DPD is a coarse-grained simulation method in which a complex molecule, such as nanoliposomes, is represented by soft spherical beads

joined with springs. The interaction is usually described through simple and pairwise-additive potentials. Similarly, to molecular dynamics simulations, particle positions and velocities in DPD are governed by the Newtonian law of motion:

$$\frac{d\mathbf{r}_i}{dt} = \mathbf{v}_i, \qquad m_i \frac{d\mathbf{v}_i}{dt} = \mathbf{F}_i, \tag{S1}$$

where $\mathbf{r}_i$, $\mathbf{v}_i$ and $\boldsymbol{m}_i$ are the position, velocity and mass of the *i*th bead, respectively, and $\mathbf{F}_i$ is the total force exerted upon it. The total force is the sum of the conservative force ($\mathbf{F}^{\boldsymbol{C}}$), random force ($\mathbf{F}^{\boldsymbol{R}}$), and dissipative force ($\mathbf{F}^{\boldsymbol{D}}$) as follow:

$$\mathbf{F}_{ij} = \sum_{i\neq j}^{N}\left[\mathbf{F}^{C}(\mathbf{r}_{ij}) + \mathbf{F}^{R}(\mathbf{r}_{ij}) + \mathbf{F}^{D}(\mathbf{r}_{ij})\right] \tag{S2}$$

The conservative force between the *i*th particle and the *j*th particle determines the thermodynamics of the DPD system and is defined by a soft repulsion:

$$\mathbf{F}_{ij}^{C} = \begin{cases} a_{ij}(1 - r_{ij})\hat{\mathbf{r}}_{ij} & r_{ij} \leq r_c \\ 0 & r_{ij} > r_c \end{cases} \tag{S3}$$

where $\boldsymbol{a}_{ij}$ is the parameter expressing the maximum repulsion between *i*th and the *j*th beads, and $\mathbf{r}_{ij} = \mathbf{r}_i - \mathbf{r}_j$, $r_{ij} = |\mathbf{r}_{ij}|$, $\hat{\mathbf{r}}_{ij} = \mathbf{r}_{ij}/r_{ij}$ is the unit vector denoting the direction from bead *i* to *j*. $\boldsymbol{r}_c$ is a cut-off radius, and it gives the extent of the interaction range between a pair of beads. The other two forces in Eq. (S2) are the random force ($\mathbf{F}^{\boldsymbol{R}}$), which is given as follows:

$$\mathbf{F}_{ij}^{R} = \sigma\omega^{R}(r_{ij})\xi_{ij}\hat{\mathbf{r}}_{ij} \tag{S4}$$

and the dissipative force ($\mathbf{F}^{\boldsymbol{D}}$):

$$\mathbf{F}_{ij}^{D} = -\gamma\omega^{D}(r_{ij})[\mathbf{r}_{ij}\cdot\mathbf{v}_{ij}]\hat{\mathbf{r}}_{ij} \tag{S5}$$

In Eq. (S4), $\boldsymbol{\sigma}$ is the amplitude of the noise. $\boldsymbol{\xi}_{ij}$ is a random number between 0 and 1 and is subject to a uniform distribution for simplicity; it is statistically independent from the pair of beads. In Eq. (S5), $\mathbf{v}_{ij} = \mathbf{v}_i - \mathbf{v}_j$ is the difference between the velocity of the *i*th bead and the *j*th bead, $\boldsymbol{\gamma}$ is the friction coefficient. The $\boldsymbol{\omega}^{\boldsymbol{R}}$ and $\boldsymbol{\omega}^{\boldsymbol{D}}$ are weight functions; the combination of the dissipative and random forces leads to a thermostat that conserves the total momentum of the system. The magnitude of the dissipative and stochastic forces are related through the fluctuation–dissipation theorem [4]:

$$\omega^{D}(r_{ij}) = [\omega^{R}(r_{ij})]^2 = max\left\{\left(1 - \frac{r_{ij}}{r_c}\right)^2, 0\right\} \tag{S6}$$

where $\boldsymbol{r}_c$ is a cut-off distance. At interparticle distances larger than $\boldsymbol{r}_c$, all forces are equal to zero. This simple distance dependence of the forces, which is a good approximation to the one obtained by spatially averaging a van der Waals–type interaction, allows one to use relatively large integration time steps. The strengths of the dissipative and random forces are related in a way that keeps the temperature internally fixed, $\boldsymbol{k}_B\boldsymbol{T} = \frac{\sigma^2}{2\gamma}$; $\boldsymbol{k}_B$ being Boltzmann's constant and T the temperature. The natural probability distribution function of the DPD model is that of the canonical ensemble, where *N* (the total particle number), *V* (Volume), and *T* (Temperature) are kept constant. The equations of motion are solved using the velocity Verlet algorithm adapted to DPD [5].

In this work, both the chains of the chitosan polymer and the molecules of lecithin and capsaicin are connected by a harmonic spring as follows

$$\mathbf{F}_{ij}^{S} = -k_s(r_{ij} - r_0)\hat{\mathbf{r}}_{ij} \tag{S7}$$

Where the spring constant is $\boldsymbol{k_s}$ and the equilibrium distance is $\boldsymbol{r_0}$ [6]. Using the same harmonic model, we control the angle between every three beads and the equation for this type of bond is

$$\mathbf{F}^{\boldsymbol{\theta}}_{\boldsymbol{ijk}} = -\boldsymbol{k_\theta}\left(\boldsymbol{\theta_{ijk}} - \boldsymbol{\theta_0}\right)\widehat{\boldsymbol{\theta}}_{\boldsymbol{ijk}} \quad \text{(S8)}$$

Where $\boldsymbol{k_\theta}$ is the spring constant, $\boldsymbol{\theta_{ijk}}$ is the angle between $\boldsymbol{i}$-$\boldsymbol{j}$-$\boldsymbol{k}$ particles and $\boldsymbol{\theta_0}$ is the equilibrium angle. For simplicity, conservative interaction parameters for each one components are listed in Table S1. The interaction parameters have been obtained using the group contribution method [7] based on the solubility of each bead and following the standard technique for parametrizing the DPD interactions [8].

Finally, two fundamental properties were used namely, the radial distribution function, $\boldsymbol{g(r)}$, and the potential mean force (PMF), $\boldsymbol{W_{PMF}(r)}$. We focus here on the latter, which is an effective pair interaction that provides important thermodynamic information about many – body systems. It can be obtained from the radial distribution functions, $\boldsymbol{g(r)}$, through the relation [9]:

$$W_{PMF}(r) = -k_B T \ln[g(r)] \quad \text{(S9)}$$

A. Models

The exact division of capsaicin, lecithin and chitosan molecules is presented in next figure S1.

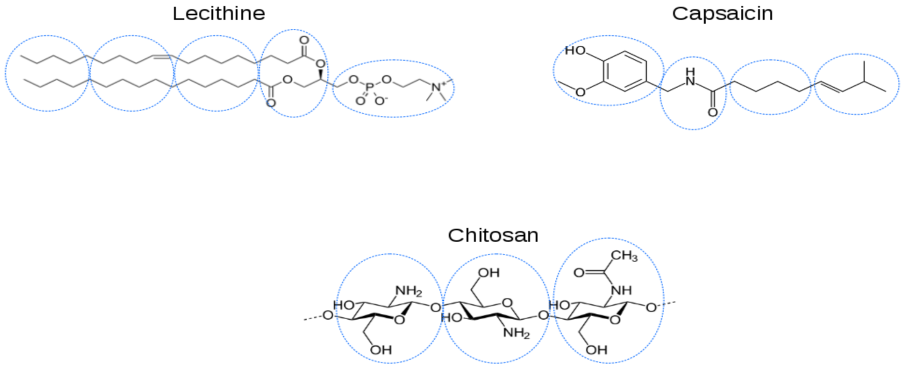

Figure S1. (Color online). Construction of beads in every molecule.

The matrix of interaction parameters $\boldsymbol{a_{ij}}$ according to Eq. S3 between every group shown in figure S1 is presented in the next table.

Table S1. Interaction matrix $\boldsymbol{a_{ij}}$. The labels in this table are according to the description of figure 1 of the original article.

	L1	L2	L3	A	G	C1	C2	C3	W
**L1**	78.33								
**L2**	80.25	78.33							
**L3**	95.21	85.85	78.33						
**A**	80.67	85.72	103.82	78.33					

**G**	82.05	87.15	103.43	78.68	78.33				
**C1**	78.34	79.51	89.85	80.89	82.35	78.33			
**C2**	89.20	93.48	103.95	84.73	83.14	89.50	78.33		
**C3**	85.72	81.48	78.34	93.79	98.29	85.35	103.47	78.33	
**W**	89.25	92.79	101.21	83.41	80.98	89.49	78.62	100.83	78.33

Parameters of the intramolecular forces are shown follows; the corresponding parameters of bonding forces are: for all molecules $\boldsymbol{r_0 = 0.7}$ and $\boldsymbol{k_s = 100}$ [6]. Parameters corresponding to binding forces are for lecithin molecules are $\boldsymbol{\theta_0 = 170.0}$ and $\boldsymbol{k_\theta = 50.0}$. For chitosan are $\boldsymbol{\theta_0 = 118.5.0}$ and $\boldsymbol{k_\theta = 10.0}$, finally for capsaicin are $\boldsymbol{\theta_0 = 175.0}$ and $\boldsymbol{k_\theta = 10.0}$. The angles $\boldsymbol{\theta_0}$ are taken of molecular structures, from representative atoms in every coarse–graining group.

Others details of our simulations are $\boldsymbol{k_B T = 1.0}$, time step $\boldsymbol{\Delta t = 0.03}$, mass $\boldsymbol{m = 1.0}$ and $\boldsymbol{r_C = 1.0}$. The parameters **σ** y γ of random and dissipative forces are equal to 3.0 and 4.5 respectively. All simulations performed 50 blocks of $\boldsymbol{1 \times 10^5}$ steps to reach a total of $\boldsymbol{5 \times 10^6}$ steps or 24 μs. The density of all systems are chose as 3.0 and the total number of particles in each simulation is 150000. All simulation parameters are in DPD units.

For fix the number of lecithin molecules that made a nanoliposome, we run an extra set of simulations, these simulations consist in change the concentration of lecithin molecules in the liposome structure. The chosen concentrations were: $\boldsymbol{\chi_{LC} = 0.48,\ 0.60,\ 0.73, \text{and } 0.85\ \text{M}}$, where the *LC* subscript refers to lecithin molecules. Density maps of these simulations shown is figure S2.

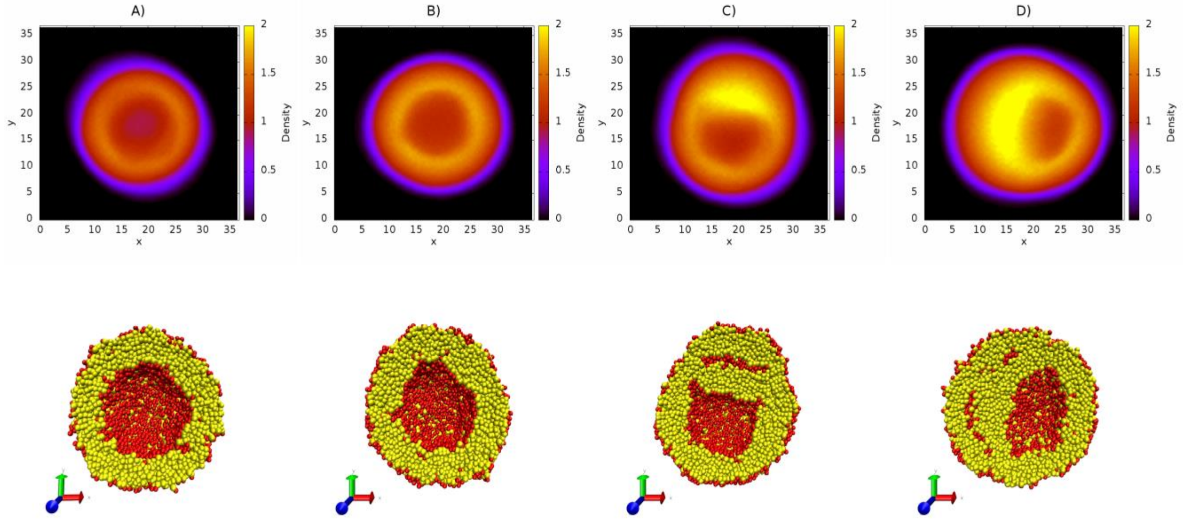

Figure S2. (Color online). Initial configuration of nanoliposome. A snapshot of the initial configuration Density maps of lecithin at different concentrations. A) 3929 lecithin molecules $\chi_{LC} = 0.48$ M. B) 4929 lecithin molecules $\chi_{LC} = 0.60$ M. C) 5929 lecithin molecules $\chi_{LC} = 0.73$ M. D) 6929 lecithin molecules $\chi_{LC} = 0.85$ M.

We use these results for choose the ideal concentration of lecithin. The concentration chosen is $\boldsymbol{\chi_{LC} = 0.60\ \text{M}}$, the reason is because in the case A) the density of lecithin is low and there is a risk of the membrane breaking and in cases C) and D) the density of lecithin is very high such that the aqueous core is smaller and the structure of liposome is deformed.

The density profiles of capsaicin and lecithin help us to estimate the mean size of nanoliposome and the encapsulation efficiency. In the figure S3 we show the density profiles only for the case of χ_{CS} = 6mM and χ_{CP} = 30mM. The way to obtain these properties is to taken the average of density profile in the *x*, *y* and *z* coordinates and measure when the density begins to increase and when the density newly is close to zero and compute the difference. This difference is taken as mean size of nanoliposome.

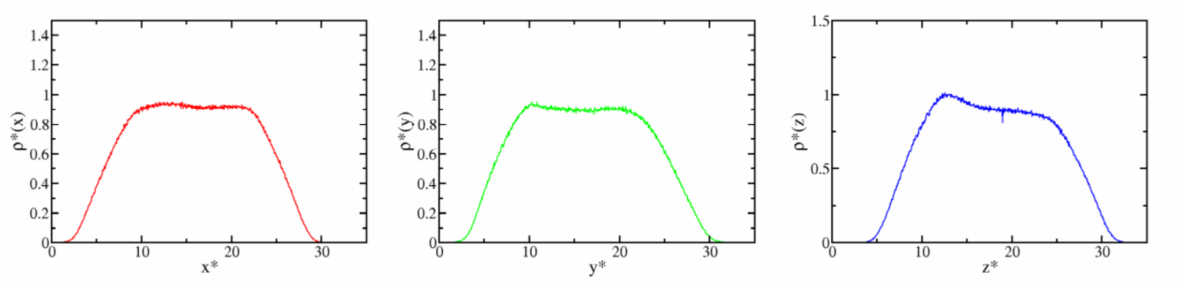

Figure S3. (Color online). Density profiles of lecithin in the coordinates *x (red)*, *y (green)* and *z (blue)* starting in left to right

For the efficiency of encapsulation is need to integrate a density profile of capsaicin for obtain the number of molecules inside the nanoliposome and applicate the equation of encapsulation efficiency (EE). See the discussion in the main text about the calculation of the EE. Density profiles of capsaicin is shown in the figure S4.

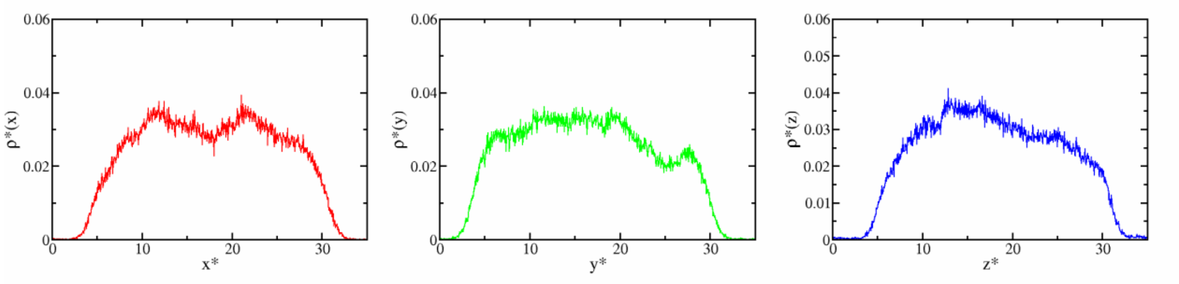

Figure S4. (Color online). Density profiles of capsaicin in the coordinates *x (red)*, *y (green) and z (blue)* starting in left to right.

References

1. Hoogerbrugge, P.J.; Koelman, J.M.V.A. Simulating microscopic hydrodynamic phenomena with dissipative particle dynamics. *Europhys. Lett.* **1992**, 19, 155-160. https://doi.org/10.1209/0295-5075/19/3/001.
2. Murtola, T.; Karttunen, M.; Vattulainen, I. Systematic coarse graining from structure using internal states: Application to phospholipid/cholesterol bilayer. *J. Chem. Phys.* **2009**, 131, 08B601. https://doi.org/10.1063/1.3167405.
3. Balderas Altamirano, .M.A.; Pérez, E.; Gama Goicochea, A. On Finite Size Effects, Ensemble Choice and Force Influence in Dissipative Particle Dynamics Simulations. C.J. Barrios Hernández et al. (Eds.): CARLA 2016, CCIS 697, High Performance Computing (pp. 314–328), Springer International Publishing.
4. Español, P.; Warren, P. Statistical mechanics of dissipative particle dynamics. *Europhys. Lett.* **1995**, 30, 191–196. https://doi.org/10.1209/0295-5075/30/4/001.
5. Vattulainen, I.; Karttunen, M.; Besold, G.; Polson, J.M. Integration schemes for dissipative particle dynamics simulations: From softly interacting systems towards hybrid models. *J. Chem. Phys*. **2002**, 116, 3967–3979. https://doi.org/10.1063/1.1450554.
6. Gama Goicochea, A.; Romero-Bastida, M.; López-Rendón, R. Dependence of thermodynamic properties of model systems on some dissipative particle dynamics parameters. *Mol. Phys*. **2007**, 105, 2375–2381. https://doi.org/10.1080/00268970701624679.

7. Ravindra, R.; Krovvidi, K.R.; Khan, A.A. *Carbohyd. Polym.* **1998**, 36, 121–127. https://doi.org/10.1016/S0144-8617(98)00020-4.
8. Groot, R.B.; Warren, P.B. *J. Chem. Phys.* **1997**, 107, 4423–4435. https://doi.org/10.1063/1.474784.
9. Roux, B. The calculation of the potential of mean force using computer simulations. *Comp. Phys. Comm.* **1995**, 91, 275–282. https://doi.org/10.1016/0010-4655(95)00053-I.

© 2018 by the authors. Submitted for possible open access publication under the terms and conditions of the Creative Commons Attribution (CC BY) license (http://creativecommons.org/licenses/by/4.0/).
